# A Spectrophotometric Assay for Robust Viability Testing of Seed Batches Using 2,3,5-Triphenyl Tetrazolium Chloride: Using *Hordeum vulgare* L. as a Model

**DOI:** 10.3389/fpls.2017.00747

**Published:** 2017-05-16

**Authors:** Laura Lopez Del Egido, David Navarro-Miró, Victor Martinez-Heredia, Peter E. Toorop, Pietro P. M. Iannetta

**Affiliations:** ^1^Ecological Sciences, James Hutton InstituteDundee, United Kingdom; ^2^Department of Earth and Environmental Science, University of PaviaPavia, Italy; ^3^Comparative Plant and Fungal Biology Department, Royal Botanic GardensKew, United Kingdom

**Keywords:** formazan, *Hordeum vulgare*, seed-testing, seed viability, tetrazolium, TTC

## Abstract

A comparative analysis was carried out of published methods to assess seed viability using 2,3,5-triphenyltetrazolium chloride (TTC) based assays of seed batches. The tests were carried out on seeds of barley (*Hordeum vulgare* cv. Optic) as a model. We established that 10% [w/v] trichloroacetic acid (TCA)/methanol is superior to the acetone and methanol-only based methods: allowing the highest recovery of formazan and the lowest background optical density (OD) readings, across seed lots comprising different ratios of viable and dead seeds. The method allowed a linear-model to accurately capture the statistically significant relationship between the quantity of formazan that could be extracted using the method we developed and the seed temperature-response, and seed viability as a function of artificially aged seed lots. Other quality control steps are defined to help ensure the assay is robust and these are reported in a Standard Operating Procedure.

## Introduction

The tetrazolium (2,3,5-tryphenyl tetrazolium chloride, TTC) test to assess seed viability from cut single seeds was originally developed by Lakon (1949) as a rapid (1–2 days) method to replace germination based assessments which could take several weeks. The test relies on the reduction of the colourless and water soluble 2,3,5-triphenyl-2H-tetrazolium chloride (TTC) to an insoluble red compound (formazan). This reduction occurs as a consequence of hydrogen ions donated to the TTC upon dehydrogenase activity in metabolically active tissues, such as the seed embryo (Junillon et al., 2014). Consequently, seed viability is usually determined using a topographical method (visual observation) to characterise the pattern and intensity of formazan staining pattern and the intensity of coloration for individual seed embryos (Copeland and McDonald, 2001). The TTC-test is commonly used to assess the viability of seeds that have failed to germinate (Parreño-de Guzman et al., 2011; Brar et al., 2013; Rami and Patel, 2014).

There are internationally recognised methods for TTC seed viability testing (International Seed Testing Association (ISTA), 2014), which can be as reliable as germination tests for seeds of some species and purposes (*Rubia fruticosa* Ait., Marrero et al., 2007); orchids (Custódio et al., 2016); grasses (Soares et al., 2016); and *Cucumis anguria* L. (Paiva et al., 2017). While the topographical method requires extensive experience to achieve an accurate interpretation, germination tests are still commonly used (Mastouri et al., 2010; Moreira et al., 2010; Van Treuren et al., 2013; Ntuli et al., 2015). In addition, there is confusion in the literature regarding seed pre-treatments and choice of extraction protocols and especially the solvent used to extract the formazan which is formed.

TTC testing is also used to test the viability and intensity of metabolic activity in other types of biologically active specimens including plant parts e.g., fine-roots of Norway spruce (*Picea abies* L. Karst, Ruf and Brunner, 2003), plant leaves and stems; fungi (*Aspergillus niger*, Ghaly and Mahmoud, 2007) and *Corylus avellana* pollen long term stored viability (Novara et al., 2017). In some cases, the formazan produced during the staining period is extracted in a liquid, the optical density of which is quantified using spectrophotometry (using light of wavelengths at or close to 484 ηm). However, formazan extraction is still not widely used to determine seed viability although there have been some attempts to standardise and develop the assay for this purpose (Harty et al., 1972; Norton, 1985; Zhao et al., 2010), and a comparison of the protocols (and those developed here) are provided in Table 1.

**Table 1:** **A summary of methods examined to assess seed viability in seed lots using the TTC-assay**.

**Method**	**Sources**	**Test species**	**Test material**	**Conditions for incubation of test material with TTC**			**Conditions for formazan extraction**			
				**Pre-treatment**	**Buffer**	**Time/Temp**.	**Homogenisation**	**Drying**	**Solvent for homogenate**	**Incubation**
I	c.f. (Harty et al., 1972)	Barley	Whole seeds	Homogenised fresh using mortar and pestle (<0.5 mm)	1% TTC in citrate-phosphate buffer (pH 7.4)	4 h/21°C^*^	None	Buchner funnel only	Methanol	15 h/30°C
II	Norton, 1985	Peas	Kernels	None	1% TTC in water	4 h (room temp.)	Homogenised (fresh in blender)	None	Acetone	None
III	c.f. (Zhao et al., 2010)	Maize	Kernels	None	0.1% TTC 50 mM Tris-HCl buffer (pH 7.6)	4 h/21°C^*^	Homogenised under liquid-N_2_ using a mortar and pestle	None	10% TCA/acetone	5 min/room temp. (21°C)
IV	This study	Barley	Whole seeds	Bleached/sterilised seeds homogenised in blender, 1 min.	1% TTC in 5 mM Potassium phosphate buffer (pH 7.2)			Buchner funnel and overnight (21°C)	10% TCA/acetone	15 h/30°C
V									10% TCA/methanol	
VI									Acetone	

*The table describes the assays as comprising two main stages which are the conditions for: (1) incubation of the test material with TTC, and; (2) formazan extraction. Methods I–III describe protocols for three methods reported in the peer-reviewed literature and these were assessed in parallel with methods IV–VI. Methods IV–VI were developed in the course of the research reported in this manuscript, and they combine aspects of methods I and III with other new steps (defined in Table 3 and proposed as a recommended “standard operating procedure”)*.

* *Denotes a modification to the TTC conditions for comparative purposes with methods V–VI. The original TTC incubation conditions for Harty et al. (1972) were 2 h, 30°C, and for Zhao et al. (2010) were 24 h, 30°C*.

In 1997, Vankus described the time-consuming limitations of the current TTC protocols (Vankus, 1997). Also, Gaspar-Oliveira et al. (2011) and Zeng et al. (2014) also disclosed time-consuming pre-conditioning and preparations steps for TTC testing. Therefore, a robust high throughput technique to test seed viability in seed batches would be a great benefit as such a method would not require time-consuming seed pre-treatments such as dehulling or dissection. In a comparative analysis of glacial acetic acid with methanol, Harty et al. (1972) established the latter as a better solvent for formazan extraction from TTC-treated milled seeds. However, we hypothesised that the seed batch process could be improved. For example, the physiological state of the test material used by Harty et al. (1972) was “natural,” and not standardised under controlled experimental conditions. Also, potentially important steps could be added such as “seed bleaching” (Peters, 2000), which can remove pigments that would otherwise have been extracted and caused high optical densities in the test extracts and control samples. The method used by Norton (1985) used whole bisected seed kernels (testa removed), which is time consuming and therefore cannot be used for high-throughput processing. Furthermore, the dissected seeds were incubated with 1% [w/v] TTC for periods of up to 4 h and formazan extraction was achieved by homogenisation in acetone (95% [v/v]). Also, it is important to note that later publications have highlighted that acetone is inferior to methanol for formazan extraction (Zhao et al., 2010). Additionally, Norton (1985) applied TTC to the dissected half-kernels: that is, the reduction potential of the whole tissue was not assessed, and imbibition of the dissected kernels on filter-paper moistened with TTC solution was advocated, which may not lead to standard treatment of the test material. Furthermore, important extraction conditions such as the homogenisation period and TTC incubation temperature were not specified. However, Norton (1985) did standardise the quantities of formazan recovered for the weight of kernels which were treated. Similarly, Zhao et al. (2010) established the recovery of formazan by homogenization and incubation of excised embryos was greater for 10% TCA:acetone [v/v] than 80% acetone:water [v/v]. As this method also required dehulling it is therefore still time consuming. Also, seed viability was assessed indirectly using the quantity of malondialdehyde (MDA; a breakdown product of lipid peroxidation, and therefore cell damage/death). That is, seed viability was not tested by germination nor had the test samples been standardised: for example, to contain known proportions of viable and dead seeds, or treated to control seed vigour.

It therefore appears that the TTC based assays currently available in the literature do not yet describe a single robust or high-throughput spectrophotometric assay to assess seed viability of seed lots. This short-coming may be linked to the profusion of information regarding the TTC assays for other types of metabolically active tissue which may have led to conflicting and sub-optimal approaches. Crucially, it should be noted that formazan production is correlated with staining time (Harty et al., 1972; Mikuła et al., 2006), TTC concentration (Steponkus and Lanphear, 1967; Harty et al., 1972; Junillon et al., 2014) and incubation temperature for seed imbibition and TCC reduction where this is done on seed extracts. Equally, the quantity of formazan extracted and exclusion of secondary compounds that may interfere with the assay depends upon the type of solvent used and the extraction protocol: such as the extent to which moisture is removed from the test material after incubation in TTC. In addition, the quantity of formazan extracted should be standardised for the weight of seed tissue which is tested. We note that the vapour pressure (evaporation rate), for each formazan-carrying solvent is highly variable with: acetone > methanol > ethanol. These solvents may therefore differentially affect the stability of optical densities recorded using the spectrophotometry for TTC-based assays. The relative capacity of solvents to directly affect the OD which is recorded seems untested. Additionally, no recommendations seem to have been made to ensure that the solvent-formazan extract should be treated to minimise evaporation during storage and reading. This manuscript therefore uses seeds of barley (*Hordeum vulgare* L. cv. Optic) in a model approach based on significant modifications and developments of Harty et al. (1972), Norton (1985) and Zhao et al. (2010). The aim of the approach was to establish a more-robust and accurate assay to quantify seed viability.

## Materials and methods

### Seed imbibition and germination

Initial tests assessed batches containing mixtures of viable (99.8% germination), and dead (by dry autoclaving at 120°C, 20 min) barley (cv. Optic) caryopses, hereafter referred to as seeds. A series of standard test samples were prepared (in triplicate) by combining viable:dead seeds to a total of 100 seeds (*ca*. 7.5 g). The inclusion of dead seeds [w/w] was either: 0, 20, 40, 60, 80, or 100%. Seeds were left to imbibe overnight between water-saturated tissue paper inside a sealed container and incubated in the dark at 20°C. Rarely, any seed with a protruding shoot-born root was removed prior to processing for treatment with TTC.

Quantification of seed germination across a temperature series (10, 15, 20, 25, and 30°C), was also performed in the dark and scored when protrusion of the first shoot-born root was evident (*ca*. 1 mm; Tillich, 2007): these conditions were also used to confirm the efficacy of dry autoclaving to kill the seeds. It should also be noted that seeds for treatment and the sterile distilled water (SDW) and SDW-pre-soaked paper-tissues for imbibition were pre-incubated at their respective treatment temperatures for 2 h.

Germination tests were performed using 6 replicates of 50 seeds *per* Petri dish (300 *per* temperature), sown on to 3 MM Whatmann paper and incubated for 12–14 h in a sealed container inside a controlled environment cabinet (in darkness) before watering with 8 mL of SDW. Germination was scored at the same hour every day for 10 days and seeds with protruded shoot-born roots were removed.

### Controlled ageing treatment

Barley seeds cv. optic were placed in glass vials inside an electrical enclosure box (catalogue number OABP303010B, Ensto UK Ltd., Southampton) sealed with a clear lid, above a solution with *ca*. 250 g LiCl added to 1 L of distilled water, producing 70% relative humidity. The seeds were placed for 2 weeks at 20°C to allow equilibration to the high humidity, and subsequently at 45°C to allow ageing. Seeds were sampled after 0, 8, 18, 25, and 31 days ageing. After each ageing period, 6 samples of 50 seeds were removed for germination testing, and 4 samples of 50 seeds for formazan extraction. The samples for formazan assay were dried on silica gel and stored at 15% RH at 15°C until extraction. Germination tests were carried out immediately by sowing the 6 replicates of 50 seeds on 1% [w/v] agar dishes. Dishes were incubated at 15°C under a 8 h photoperiod and germination was scored frequently up to 3 weeks. Germination was considered complete when emission of the first shoot-born root was detectable >1 mm. For the TTC extraction, three replicates were used in the initial tests, whereas for the controlled-aged samples, four replicates were used.

### Tetrazolium assay

The assay was performed on three replicates of 100 seeds in the case of the viable:dead seeds (1,800 seeds in total) and, on 4 replicates of 50 seeds in the case of the controlled aged seeds (1,200 seeds in total). Therefore, the volumes of solutions described below were used in the first case, for the second, the volumes used were halved. Imbibed seeds were bleached using 7 mL of 3% [v/v] hydrogen peroxide (Sigma-Aldrich, #H1009) for 10 min. before rinsing twice with SDW. Seeds were briefly blotted dry between tissue paper before grinding. The treated seeds were then homogenised by grinding for 1 min (in coffee grinder; James Martin by Wahl ZX595 Mini Grinder, 150 W), and transferred to a fresh 50 mL tube with 15 mL of 1% [w/v] TTC stock solution Sigma-Aldrich, #T8877, in 5 mM potassium phosphate buffer, pH 7.2, prepared as described in Peters (2000). The TTC stock solution was kept in the dark at 4°C as it is light-sensitive (Ghaly and Mahmoud, 2007). The samples were then incubated at room temperature for 4 h in the dark without shaking before centrifugation (5,100 rpm for 5 min, Sigma 4K-15) and the supernatant was removed. The stained tissues were rinsed twice with SDW and the residual TTC solution was eliminated by vacuum drying using a Buchner funnel.

The recovered paste was treated for formazan extraction by freezing (with liquid N_2_), and grinding in a mortar and pestle, to which 7 mL of solvent was added. The incubation conditions varied according to the methods trialled here (see Table 1), which were either: I, Harty et al. (1972), methanol (100%) for 15 h at 30°C; III, Zhao et al. (2010) 10% TCA/acetone [v/v] 5 min. at room temperature; IV, 10% TCA/ acetone; V, 10% TCA:methanol, and; VI, acetone only: the final three solvents applied using incubation conditions of 15 h at 30°C. After incubation, the samples were centrifuged (15 min, 5,100 rpm), and *ca*. 2 mL of the supernatant was transferred to a 2 mL microfuge tube and re-centrifuged (14,680 rpm for 30 min at room temperature, Sigma 1-15K).

Immediately before reading the optical density (OD) of the recovered solvent, the sample (1.5 mL) was transferred to a new microfuge tube and re-centrifuged as before. Absorbance's at 484 ηm (using at ELx800™ Absorbance Reader, BioTek® Inc.), were acquired for the technical replicates (300 μl each) dispensed into spate wells of a 96-well flat bottom “ELISA plate” (Nunc MaxiSorp®, manufacturers code 439454). The values obtained were corrected for background using the average reading for the solvent only replicates. The percentage variation between the three sub-samples was calculated with the following formula (1):

(1) $$\begin{array}{r} \text{Variation}\left(\text{\%}\right)=\frac{\left(\text{Highest OD}\right)-\left(\text{Lowest OD}\right)}{\left(\text{Highest OD}\right)}*100 \end{array}$$

### Formazan standard curve

The relationship between the optical density and formazan concentration was determined using series of standard solutions of 0, 10, 20, 40, 60, 80, and 100 mg mL^−1^ red formazan; (1,3,5-triphenyltetrazolium formazan, 90% pure, Sigma Aldrich #93145). The optical densities of the standards were also corrected against background (solvent only), before plotting the standard curve and fitting the linear-model.

### Statistical analysis

Linear models were fitted using Microsoft Excel 2010 for Windows 7. Regression analysis was performed with GenStat v14.2. Results were considered significant at *P* < 0.05.

## Results

The formazan extraction conditions of Zhao et al. (2010; 5 min. at room temperature; Table 1), recovered the least formazan in both viable and dead seed types (Table 2), probably as a function of the limited extraction time (Table 1). High and similar recoveries were achieved with acetone, 10% TCA:acetone or 10% TCA:methanol (at 15 h, 30°C; Table 2). The different solvent types gave variable OD readings as shown from the three technical replicates: variation being calculated according to formula [1]. Variability in the OD readings is also apparent from the SEs (Table 2). The extraction solution (methanol) and approach originally proposed by Harty et al. (1972) gave more stable OD readings, while Method V (10%TCA:methanol, and longer TTC incubation time), also gave stable OD readings and allowed greater formazan yields (27%), than the Harty-method. The variation in OD readings which were recorded when recovering formazan using the different solvent types (Table 2) is most likely caused as a function of their relative vapour pressures. These vapour pressures may also have influenced the consistency of extraction. This data also indicated that unstable OD readings were more likely when acetone was used for either viable or dead seed extractions. On this basis, we can exclude the use of acetone.

**Table 2:** **Formazan recovered (±SE, μg mL^**−1**^) from milled 100%-viable and -dead batches of ***Hordeum vulgare*** (cv. Optic) seed that had been incubated with TTC**.

**Method number**	**Sources**	**Extraction solvent**	**Seed type**	**Formazan recovered**	
				**μg mL^−1^**	**% Variation**
I	Harty et al., 1972	Methanol^*^	Viable	39.3 ± 3.3	8.31
			Dead	13.7 ± 0.9	6.23
III	Zhao et al., 2010	10% TCA/acetone	Viable	16.2 ± 7.0	43.32
			Dead	6.6 ± 1.5	22.70
IV	This study	10% TCA/acetone	Viable	47.0 ± 7.8	16.65
			Dead	16.6 ± 7.4	44.84
V		10% TCA/methanol^*^	Viable	45.6 ± 7.4	16.30
			Dead	13.2 ± 1.1	8.31
VI		Acetone	Viable	40.3 ± 7.9	19.61
			Dead	17.0 ± 5.8	33.88

Formazan extraction used Methods defined in order (as Table 1), with: I, c.f. Harty et al. (1972), methanol; III, c.f. Zhao et al. (2010), 10% TCA/acetone with 5 min. extraction time at room temperature (21°C); IV, modification of Zhao et al. (2010), (hatched bar); V, 10% TCA:methanol; and, VI, acetone. Also shown is the % Variation in the quantity of formazan extracted from replicates (n = 3), for each Method with either 100%-viable or -dead seeds. The data distinguish methods which provide data of low variability at the point of optical density measurement, and these are denoted “

* *.” All extractions were carried out by incubation at 30°C for 15 h with the exception of Method III which used 5 min. at 21°C. Data was not acquired for Method II (Norton, 1985), though how Method II may perform under out test conditions is illustrated using Method VI*.

It is also noted that optical densities recorded in the 100% dead-seeds samples is related to pigments removed from seeds during extraction, and the resultant extract appears yellow, not red. These values present a background OD that is unrelated to metabolism but should be taken into account when calculating formazan production. Furthermore, we advocate that seeds are bleached and sterilised with 3% hydrogen peroxide (H_2_O_2_), prior to the TTC application. This sterilising treatment excluded the possibility of TTC reduction due to microbial contaminants (Peters, 2000) and also reduced the colour of the barley seeds testa, thus suppressing the otherwise higher background-OD level (data not shown).

**Figure 1:**
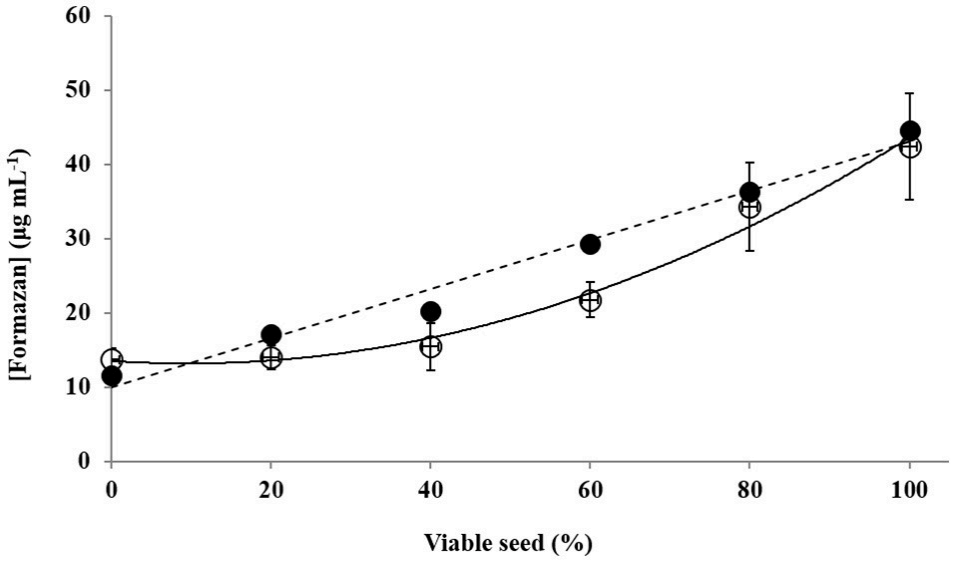
**A comparison between concentration of formazan that was extracted (±SE, μg mL^**−1**^) from the TTC-incubated seed homogenate using either Method I of Harty et al. (**1972**; °, methanol) and Method V (•, 10% TCA:methanol; see Table **1**)**. Each method allowed the fitting of polynomial- (—, solid-line) and linear- (- - -, dashed line) models, respectively.

**Figure 2:**
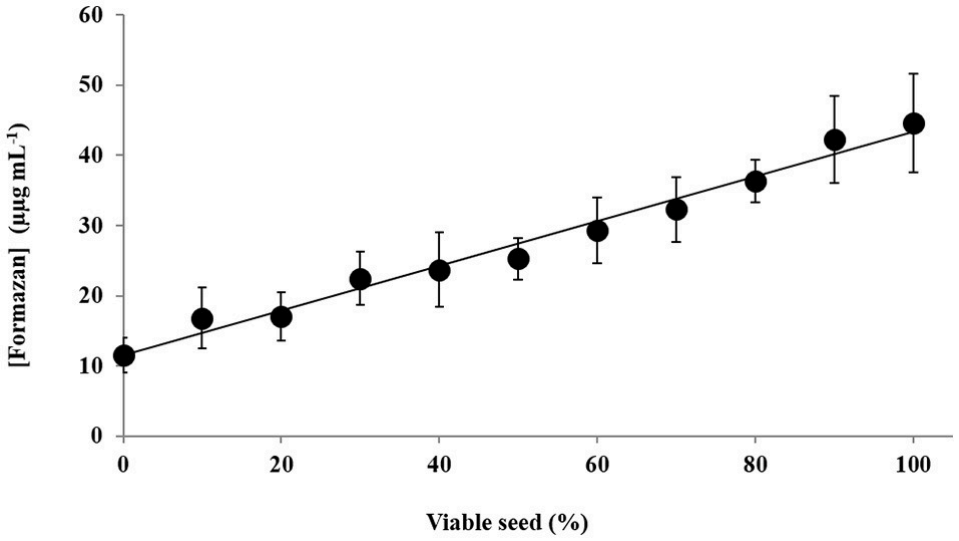
**Data acquired using Method V (10% TCA:methanol; see Table **1**) showing the quantity of formazan extracted (•, μg mL^**−1**^) plotted against the proportion of viable seeds in the test material**. The solid line shows the linear-model which was fitted.

The two methanol based methods were therefore used to assess the relationship between the concentration of formazan recovered and seed lots standardised to contain set portions of viable and dead seeds (Figure 1). The Harty-Method showed a polynomial relationship (Figure 1; *y* = 0.0037*x*^2^−0.0711*x*+13.526; *R*^2^ = 0.9851; *P* < 0.001), while Method V (10% TCA:methanol), identified a linear relationship (Figure 1; *y* = 0.3316*x*+9.9714; *R*^2^ = 0.9714; *P* < 0.001). The variation in the OD for these two methods were also compared (Table 2), and showed no significant difference. This highlighted that the Harty-method had no power to discriminate between seed batches containing 0–40% live seeds, as the curve (Figure 1), was effectively flat in that range. Using Method V the concentration of formazan (μg mL^−1^) extracted from the barley seed standards (Figure 2) was quantified using standards which fitted a linear model (*y* = 0.0272*x*−0.0166; *R*^2^ = 0.9987; *P* < 0.001, data not shown). Regression analysis of data for samples “proportion of viable barley seeds in the seed-lot tested” and “concentration of formazan” showed a statistically significant linear relationship (Figure 2; *y* = 0.0318*x* + 1.1615; *R*^2^ = 0.9804; *P* < 0.001). These linear relationships between seed viability and higher recovery of formazan (Figure 1) using Method V (10% TCA:methanol), and across all viable: dead-seed proportions (Figure 2), means that we recommend 10% TCA:methanol for formazan extraction.

**Figure 3:**
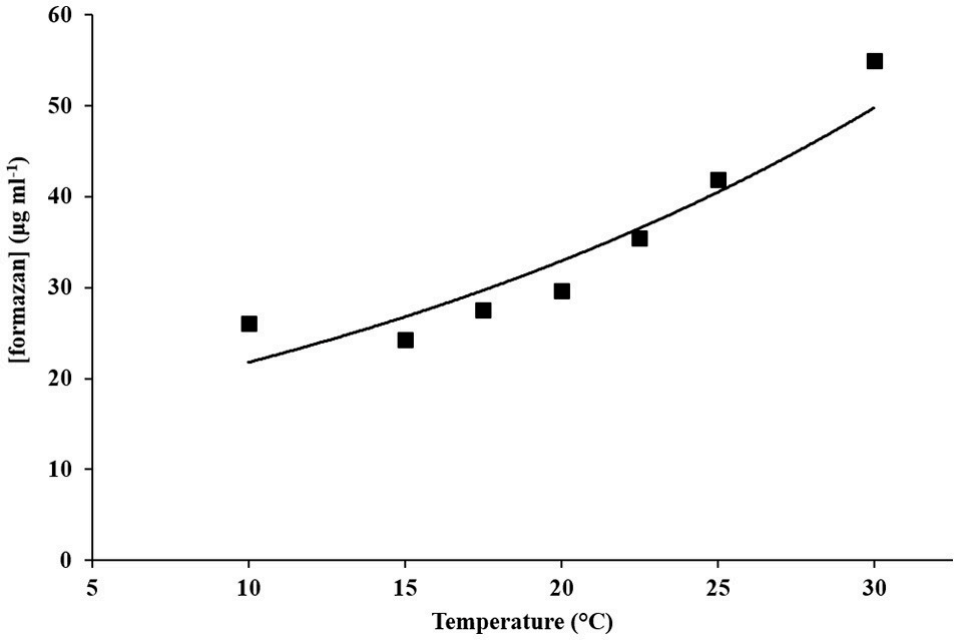
**The relationship between temperature of germination (°C) vs. average concentration of formazan extracted (■, μg ml^**−1**^ ±SE)**.

Method V was also applied to examine the relationship between the concentrations of formazan recovered from 100% viable seed lots and formazan concentration extracted from seeds whose response was controlled using a temperature series (Figure 3). This analysis found a significant linear relationship between the concentration of formazan which could be recovered and log final germination at the different temperatures (Figure 3; *y* = 14.414e^0.0413x^, *R*^2^ = 0.8646; *P* < 0.001). These results demonstrate that a linear model can also be used to predict seed temperature response (final germination) from formazan concentration over that temperature range for which their relationship is monotonic: which for the barley seeds used here was 10–30°C. At a high temperature, reactivation of metabolism in more rapid and this is reflected in a high formazan extraction. Nevertheless, at high temperatures percentage of germination is low, because of thermo-inhibition. Therefore, germination and metabolic reactivation are uncoupled events. This counter-intuitive result demonstrates the importance of temperature control.

**Figure 4:**
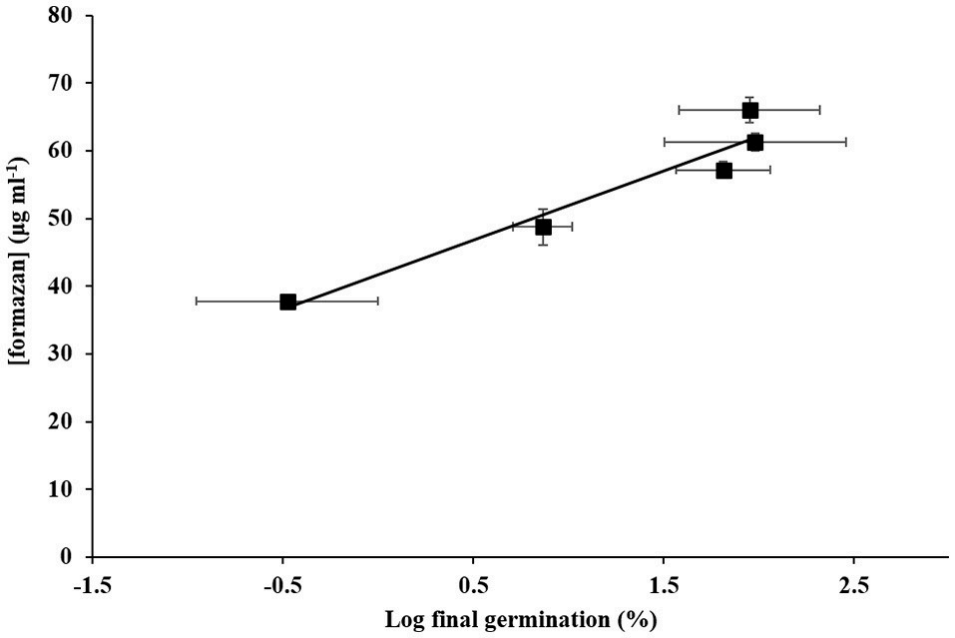
**The relationship between final germination after controlled ageing and formazan extracted: log final germination (%) vs. concentration of formazan extracted (■, μg ml^**−1**^ ±SE)**.

Method V was then applied to seeds after controlled aging, which showed a significant linear relationship between the concentration of formazan recovered and log final germination percentage of the seed batches with varying viability (Figure 4; *y* = 10.185*x* + 41.711, *R*^2^ = 0.9335; *P* < 0.001).

The data reported here highlights the risks of using acetone to test viability of bulk seed lots. A standard operating procedure is suggested and this was described in the Table 3.

**Table 3:** **A standard operating procedure for the spectrophotometric assay to test the viability of seed batches: using minimum of three technical replicates of barley (***Hordeum vulgare*** cv. optic) seed as a model**.

1. Place the seeds between water saturated tissue paper to imbibe over-night (ca. 16 h) in a sealed container.^*^
2. Remove any seeds with protruded shoot-born roots.
3. Dry the seeds and transfer a fixed weight (7.5 g, *ca*. 100 seeds), to a 50 mL container.
4. Bleach/sterilise the seeds by soaking in 7 mL 3% hydrogen peroxide (Sigma-Aldrich, #H1009) for 10 min.
5. Wash 2 times with 20 mL of sterile distilled water.
6. Dry the seeds with tissue paper before grinding in blender for 1 min (James Martin by Wahl ZX595 Mini Grinder, 150 W)
7. Transfer all the seed flour to a fresh 50 mL tube.^*^
8. Add 15 mL of TTC (Sigma-Aldrich, #T8877) in 1% [w/v] with 5mM potassium phosphate buffer, ph 7.2 (Peters, 2000).
9. Homogenise by vortexing for 15 s.
10. Incubate for 4 h in darkness at room temperature (21°C).^*^
11. Centrifuge at 5,100 rpm for 5 min. (Sigma 4K-15) and remove the supernatant^a^.
12. Suspend residue in 20 mL sterile distilled water and re-centrifuge (as 11); repeat this step.
13. Transfer the residue to filter-paper (Whatmann No.3) on a Buchner-funnel^b^.
14. Remove excess moisture from the residue under vacuum.
15. Place the entire residue into a sterile container to dry over-night (21°C).
16. Grind the whole dried residue to a powder using a mortar and pestle.
17. Re-grind the residue after the addition of liquid N_2_^c^.
18. Dry at 30°C 10 min. and decant the fine powder to new 50 mL centrifuge tubes.
19. Add 7 mL of 10% TCA (Sigma-Aldrich, #T6399) [w/v] /methanol and vortex 3 × 30 s.^*^
20. Incubate the samples overnight (15 h) at 30°C.
21. Centrifuge 15 min. at 5,100 rpm at room temperature (Sigma 4K-15).
22. Remove 2 mL of the extract and transfer to microfuge tube.
23. Centrifuge the microfuge tube for 30 min. at 14,680 rpm at room temperature (Sigma 1-15K).
24. Transfer 1.5 mL of the supernatant to fresh 1.5 mL microfuge tube^d^.
**OPTICAL DENSITY (OD) DETERMINATION**
25. Re-centrifuge 1.5 mL extract for 20 min. at 14,680 rpm at room temperature (Sigma 1-15K).
26. Dispense 300 μl (extract, controls, blanks), to separate wells of flat-bottomed ELISA plate (Nunc MaxiSorp®, manufacturers code 439454).
27. Read the optical density of each at 484 nm (using at ELx800™ Absorbance Reader, BioTek® Inc.).
28. Correct the OD s by subtracting the background (solvent-only/ TTC-untreated controls).
29. Use the average reading of the three technical replicates.
**DATA ANALYSIS**
30. Correct test data: subtract average background of the exaction solvent-only control.
31. Convert the OD to μg mL^−1^ formazan (Sigma Aldrich #93145) using the linear-model of standard samples^e^.

* *This highlights steps of the protocol which may need modified and/or standardised for seeds for other species according to their parameters such as thousand seed weight and/or the (metabolically active) embryo weight relative to that of the endosperm*.

a *Ensure all of the supernatant is removed as this may interfere with OD measurements*.

b *Ensure all the residue is transferred to the filter-paper*.

c *Ensure that there is no loss of material during grinding in the mortar*.

d *Ensure that residue is not re-suspended during pipetting*.

e *The standard samples are prepare by dissolving formazan in extraction solvent*.

This provided a detailed standard operating procedure for the 10% TCA:methanol based technique, and it represents a significant development of Harty et al. (1972). A justification of the steps involved is given in the Discussion which follows.

## Discussion

TTC based assays have proven reliable to test viability in plant tissues such as the inner bark tissues of *Quercus serrata* Murray (Shimomura and Hasebe, 2004), roots from Norway spruce (*P. abies* (L) Karst (Ruf and Brunner, 2003), grape roots (*Vitis vinifera*, Comas et al., 2000) among others. However, the use of a spectrophotometric assay for testing of seed viability and germination response to temperature is not commonly used. It would appear that the profusion of information from the use of TTC to test viability and temperature response in other fields of biology is married to a lack of unanimity among seed-based tests. It is therefore important that a standard protocol that provides greater clarity is established. Among the variables, we acknowledge that TTC incubation time may vary from 2 to 3 h (for barley, Grzybowski et al., 2012), to 24 h or more (e.g., maize, Zhao et al., 2010). In addition, TTC concentration may range from 0.1% (e.g., Zhao et al., 2010), to 1% (Harty et al., 1972; Norton, 1985), and both these variables may correlate with formazan production (Steponkus and Lanphear, 1967; Harty et al., 1972; Mikuła et al., 2006). Thus, in order to obtain an optimal formazan production and facilitate the high-throughput nature of the assay: incubation of pre-soaked ground seeds in 1% [w/v] TTC in potassium phosphate buffer for 4 h provided the optimal balance of rapidity with high levels of formazan production. The pH of the TTC solution and the temperature at which it was administered was also standardised to improve the reliability, reproducibility and accuracy of the method. The Tetrazolium Testing Handbook (Peters, 2000), establishes a range of acceptable pH's which range from 6.5 to 7.5, and temperatures ranging from 20 to 40°C, and the standard protocol described here falls within the limits recommended.

The formazan produced in the staining reaction is water-insoluble and so the moisture content of the samples may affect the extraction efficiency. Harty et al. (1972) used the Buchner funnel to reduce the moisture after washing the samples. However, the moisture remains within the residue and this may influence the extraction efficiency. For this reason, the method we describe here also involved drying the residue after filtration. Additionally, the efficiency of formazan extraction is also dependent on the extent to which the stained tissue is disrupted and so grinding tissues after solvent application is recommended (Ruf and Brunner, 2003). Therefore, we explain that the stained tissues were re-ground to a very fine powder using a mortar and pestle with liquid-N_2_ prior to the addition of solvent.

The choice of extraction-solvent used is also very important and the most commonly reported solvent is ethanol (Ruf and Brunner, 2003; Shimomura and Hasebe, 2004; Ghaly and Mahmoud, 2007), followed by methanol (Harty et al., 1972), acetone (Norton, 1985) and 10% TCA/acetone (Zhao et al., 2010). However, while ethanol has the lowest vapour pressure supporting low variability during spectrophotometrics, it is also least efficient at extracting formazan (Harty et al., 1972; Zhao et al., 2010). While this inefficiency may be compensated using heat (above 80°C; Steponkus and Lanphear, 1967; Stattin and Lindström, 1999; Verleysen et al., 2004; Mikuła et al., 2006), the method also releases compounds which lead to high background and/or high ODs at 484 ηm which are not related to formazan production (Ruf and Brunner, 2003). As a consequence ethanol was discarded as a suitable solvent.

In the previous studies (Harty et al., 1972; Zhao et al., 2010), TTC reduction was also assessed in seed-batches of mainly viable seeds. We therefore considered it important that this study assess the accuracy and reliability of the methods by examining variation between samples containing high proportions of dead seeds too. Towards that end, it was also necessary that the presence of pigments which may confound the accuracy of the test be limited and the use of seed-bleaching also helped in this respect too. The presence of TCA in the extraction solvent appears to help considerably in this respect, most likely as a function of its capacity to help precipitate proteins and degrade other polar particulates that would otherwise have reduced the optical densities recorded (Figure 1). Most significantly, we found that the method proposed by Harty et al. (1972; methanol only), cannot distinguish between seed-batches containing <40% viable seeds (Figure 1). Efficient TTC reduction was demonstrated to represent seed viability of aged seeds on a logarithmic scale, allowing the use of this assay in predicting viability of unknown samples due to ageing.

The findings described here also demonstrate that reduction of TTC is dependent upon seed incubation temperature, and that reduced formazan production may be the result of either an ineffective temperature control or/and reduced viability. However, the choice of barley seeds and the cultivar Optic in particular is not insignificant in this regard. Optic is a popular variety of choice by the growers and whisky distillers for its capacity for complete and uniform germination (99.9% viability for the batch used here), which are essential attributes for cropping and malting (respectively), and is used as a standard control variety to improve this crop for the brewing and distilling industries (c.f. Booer and HGCA, 2001; Koliatsou and Palmer, 2003). The consistent performance of cv. Optic in seed tests reported here, is also reflected in response curves to the temperature treatments that were imposed (Figure 3). This illustrates that the reduced formazan staining is a function of incubation temperature due to: (1) delayed development of metabolic activity including dehydrogenase enzyme activity at lower temperatures, and; (2) slower imbibition at lower temperatures, resulting in delayed development of metabolic activity (e.g., Patanè et al., 2006).

We conclude therefore that 10% TCA:methanol appears superior to any acetone and methanol-only based methods. The 10% TCA:methanol (Method V), based method we describe (Table 3) allowed: (1) the highest recovery of formazan; (2) the lowest levels of background stain which was detected at 484 ηm, and especially in samples containing high levels of dead seeds; (3) minimum variation between technical replicates that could occur on spectrophotometric recording; (4) the fit of a linear-model to accurately capture the relationship between the levels of formazan extracted and seed viability, and; (5) allowed a log-linear model to accurately capture the relationship between the level of formazan extracted and germination in response to temperature.

The seed-batch method proposed here requires as little as 5 of manual work for 20 samples, independent of the number of seeds in each sample. In a standard TTC assay where seeds are cut in half to assess viability of the embryo, up to 1 h is required *per* sample of 100 seeds (M. Marin, personal communication, November 30, 2016). However, this time may vary due to seed size, quality of the seed lot, and the individual performing the assessment. Furthermore, the method presented here it is objective and does not need of specialised training, as OD of extracted formazan indicates the viability of the seed batch. Additionally, dissected seeds do not need to be assessed under a microscope. Thus, the method we present has the potential to be less laborious, and provides an objective assessment based on a large number of seeds.

Future work should develop the improved method described here to test the utility of the method to predict the viability of seed batches for a greater variety of species and seed types. In the case of small seeds, sufficient weight (numbers) may be required to obtain a sufficient quantity of formazan, even in the minimum volume necessary (60 μl), for OD measurements in an ELISA-plate reader. On this basis, we highlight that the seed weight (number) volumes used may need to be standardised relative to important seed parameters such as thousand seed weight, or metabolically active embryo to endosperm ratio.

## Author contributions

LL performed the final assessment of the method we present and led the final drafting of the manuscript. Her efforts have proven the utility of our method to assess seed viability, temperature response and seed vigour in aged seeds. DN carried out the laboratory work and methodological development on live and dead seeds, and drafted the initial version of the manuscript. His work highlighted the confounding effects of extraction protocol, and in particular solvent choice. VM is a research assistant who had a working knowledge of barley seed testing and supported DN in the execution of the laboratory based tasks. Victor also helped develop the optimised standard operating procedure (SOP). PT is a molecular seed biologist who helped conceive the original idea in academic discussions with PPMI, and was pivotal in developing and conceptualisation the data for peer-review. PI conceived the original idea for this project in discussions (with PT). He was the Principal Investigator who led and oversaw the laboratory work, and helped finalise the manuscript and SOP. All authors contributed to the writing of the manuscript, approved the final version, and agree to be accountable to all aspects of the work.

### Conflict of interest statement

The authors declare that the research was conducted in the absence of any commercial or financial relationships that could be construed as a potential conflict of interest.
